# Factors related to the frequent use of emergency department services in Korea

**DOI:** 10.1186/s12873-023-00808-8

**Published:** 2023-06-29

**Authors:** Eun Deok Cho, Bomgyeol Kim, Do Hee Kim, Sang Gyu Lee, Suk-Yong Jang, Tae Hyun Kim

**Affiliations:** 1 National Emergency Medical Center, National Medical Center, 245 Eulji-Ro, Jung-Gu, Seoul, 04564 Republic of Korea; 2 Department of Public Health, Graduate School, Yonsei University, 50-1 Yonsei-Ro, Seodaemun-Gu, Seoul, 03722 Republic of Korea; 3 Department of Preventive Medicine, College of Medicine, Yonsei University, 50-1 Yonsei-Ro, Seodaemun-Gu, Seoul, 03722 Korea; 4 Department of Healthcare Management, Graduate School of Public Health, Yonsei University, 50-1 Yonsei-Ro, Seodaemun-Gu, Seoul, 03722 Korea

**Keywords:** Emergency medical center, Emergency department, Frequent users, Emergency service, Regional differences

## Abstract

**Background:**

Frequent Emergency Department (ED) visitors are identified by the policymakers to reduce avoidable ED visits and lessen the financial and operational burden. This study aimed to identify the factors related to the frequent use of ED services.

**Methods:**

This nationwide, cross-sectional observational study was conducted using information obtained from the 2019 National Emergency Department Information System (NEDIS) database. Frequent ED users were defined as patients with four or more ED visits a year. We performed multiple logistic regression analyses to verify the relationship among sociodemographic characteristics, residential characteristics, clinical characteristics, and frequency of ED visits.

**Results:**

Among 4,063,640 selected patients, 137,608 patients visited the ED four or more times a year (total number of visits = 735,502 times), which accounted for 3.4% and 12.8% of the total number of ED users and ED visits, respectively. A high ED visit frequency was associated with male sex, age < 9 or ≥ 70 years, Medical Aid (based on the insurance type), lower number of medical institutions and beds compared with that of the national average, and conditions, such as cancer, diabetes, renal failure, and mental illness. A low ED-visit frequency was associated with residence in regions vulnerable to emergency medical care and regions with high income. The possibility of frequent ED visits was high for patients with level 5 severity (non-emergent) and those with an increased need for medical treatment, including older patients and patients with cancer or mental illness. The possibility of frequent ED visits was low for patients aged > 19 years with level 1 severity (resuscitation).

**Conclusions:**

Health service accessibility factors, including low income and medical resource imbalance, were associated with frequent ED visits. Future large-scale prospective cohort studies are warranted to establish an efficient emergency medical system.

## Background

Frequentusers of emergency department (ED) services have been generating interest in recent years [1]. Internationally, frequent ED users comprise 3–8% of all the patients visiting the ED and 67% of all ED visits over a given period (usually 1 year) [2]. In Korea, 3.1% of ED visitors were identified as frequent ED users, denoting that these patients visited EDs more than four times per year, and such visits accounted for 14% of all ED visits in 2009 [3].

Frequent ED visits lead to substantial healthcare costs [2, 4]. Moreover, they decrease ED efficiency, contribute to ED overcrowding, and can result in the redirection of services from urgent cases [2, 5]. Therefore, policymakers and researchers have been trying to find ways to improve relevant services to individuals who must use the emergency care system—not those who use EDs by choice—at a higher rate [6].

Frequent ED users may receive a suboptimal quality of care since the care provided may be fragmented, episodic, and poorly coordinated [2, 7]. Additionally, physicians may be biased and less empathetic toward frequent ED users [8]. The frequent use of ED services may sometimes be inappropriate and non-urgent [9]. Accordingly, the uncoordinated acute care received by frequent ED users can be less effective compared with the effectiveness of typical ED or primary care [2].

Several studies have been conducted on frequent ED visits worldwide. Cross-sectional studies have demonstrated that sociodemographic, clinical, and health system-level factors contribute to frequent ED visits [10]. Specifically, minority race, low educational attainment, low income, public insurance, usual sources of outpatient medical care (other than the ED), high usage of outpatient health care resources, and poor physical and mental health are associated with an increased probability of frequent ED use [3, 11–13].

Predicting and identifying the frequent ED users could help formulate target interventions for addressing unmet health and social needs while simultaneously reducing ED use [1]. The characteristics of frequent ED users may vary according to the country as well as the location, size, and role of a hospital [12]. However, most studies have used medical records or sample data from one ED, with few studies using data from multiple EDs [12, 14–16].

To the best of our knowledge, there have been no national population-based studies on factors related to frequent ED use in Korea. Accordingly, there is a need for multi-regional and multi-departmental studies to identify the characteristics and relevant factors related to frequent ED users. Therefore, we aimed to identify the characteristics of frequent ED users at the emergency medical center level and factors related to their frequent visits based on nationally representative and population-based data in Korea.

## Methods

### Aim of the study

To identify the characteristics of frequent ED users at the emergency medical center level and factors related to their frequent visits based on nationally representative and population-based data in Korea.

### Study design and setting

This nationwide, cross-sectional observational study was conducted using information obtained from the National Emergency Department Information System (NEDIS) database, which is managed by the Ministry of Health and Welfare and comprises nationwide data on ED visitors in Korea.

### Participants and data source

We used NEDIS data collected from January 2019 to December 2019. The NEDIS contains data regarding patient demographics and clinical information, including age, sex, visit route, the Korean Triage and Acuity Scale (KTAS) level, vital signs, discharge outcomes, and diagnosis in ED [17].

The final analysis dataset was created by merging the public information from areas underserved by emergency services. Emergency medical centers in Korea are categorized into four types: regional emergency medical centers, local emergency medical centers, regional emergency medical institutions, and specialized emergency centers [18]. The structure of the system is designed to be a sequential emergency medical delivery system where the most severe emergency patients are treated in regional centers, while moderate or mild emergency patients are treated in local centers and institutions [18]. Moreover, under the structure, special emergency diseases are handled by specialized emergency centers (e.g., children, poisoning, burns) providing applicable treatment [18]. Since 2016, relevant authorities have made considerable efforts so that the system as a whole can achieve the goal of regionalization of emergency medical care [19]. Regional emergency medical centers strive to improve health outcomes for emergency patients by making better use of resources within the region [20]. Meanwhile, local emergency medical centers—usually with 35 beds and serving approximately 30,000 patients per year—aim to provide emergency services to patients living in rural or remote areas [21].

Areas with underserved emergency medical services are those in which more than 30% of the local population cannot reach the local emergency medical center within 30 min or the regional emergency medical center within 1 h. These locations were identified as those revised and promulgated by the Ministry of Health and Welfare of Korea in 2019 under Article 12, paragraphs 2 and 3 of the Public Health and Medical Services Act [22].

We included patients who visited one or more of the EDs of 38 and 124 regional and local emergency medical centers, respectively, which mainly provide medical treatment for emergency patients and have similar variables registered in the NEDIS database. We included 4,063,640 ED users after excluding 183,010 cases with missing patient residence or unknown data.

### Variables and measurements

#### Dependent variables

Frequent ED users are patients who visit the ED on multiple occasions [16]. We categorized patients according to their ED utilization level. We defined frequent ED users as patients with four or more ED visits a year. There is currently no established definition of high utilization; however, ≥ 4 ED visits in 1 year is a commonly used threshold [11, 13, 16]. However, given the nature of the data constructed for each institution, cases where the same patients who visited another ED may have been omitted.

#### Independent variables

Sociodemographic characteristics: Sociodemographic characteristics included sex (male and female), age (0–70 years), place of residence (Seoul, Busan, Daegu, Incheon, Gwangju, Daejeon, Ulsan, Sejong, Gyeonggi, Gangwon, Chungcheong, Jeolla, Gyeongsang, and Jeju), and insurance type (National Health Insurance [NHI; regional + employer-provided], automobile insurance, industrial accident insurance, Medical Aid [Types 1 and 2], general insurance, or others). To note, the names of the regions are original names under the Korean official administrative regional division. South Korea’s health insurance system is a public and single-payer system. With the enactment of the NHI Act in 2000, all insurers were integrated under a single insurer [23]. The NHI covers 97% of the population, and the remaining 3% is covered by the Medical Aid program [24]. Unlike NHI and Medical Aid, which are provided to all Koreans through the government, automobile insurance is a private insurance service that automobile owners must subscribe to for vehicle accident coverage [25]. Industrial accident insurance provides prompt and fair compensation for employees affected by occupational accidents through industrial accident compensation insurers [26].

Place of residence characteristics: These characteristics included information regarding the status of emergency medical services (underserved or not), the number of emergency medical institutions and number of beds available in the area, and the regional income decile. Areas lacking emergency medical services were identified from those announced by the Ministry of Health and Welfare [19, 27]. Regarding the number of emergency medical institutions and beds, data were categorized using the following criteria: the number of emergency medical institutions per million residents in the city (Si), county (Gun), and district (Gu) of residence, and whether the number of beds in institutions above the hospital-clinic level was above or below that of the national average. Regarding regional income deciles, we divided the average earned income per resident of the city (Si), county (Gun), and district (Gu) into 10 deciles, from the lowest income level to the highest.

Practice-related characteristics: These variables included the presence or absence of five diseases (cancer, high blood pressure, diabetes, renal failure, and mental illness) and the results of acuity classification. Disease prevalence was classified into present or absent based on whether the disease was characterized by at least 20 main symptoms presented in the NEDIS database, based on the Korean Standard Classification of Diseases-7 code. The result of the acuity classification was processed using the KTAS level [28]. The KTAS level is divided into five levels, with the lower levels indicating higher clinical severity, as follows: level 1 necessitates top priority for care and indicates life-threatening conditions, including cardiac arrest, severe respiratory failure, and loss of consciousness, requiring immediate treatment; level 2 indicates potentially life-threatening conditions, including myocardial infarction, cerebral hemorrhage, and cerebral infarction, requiring rapid treatment; level 3 indicates conditions that can eventually progress to cause serious complications; level 4 represents conditions that require treatment or reassessment within 1 to 2 h and is associated with age, pain level, and the likelihood of complications; and level 5 indicates an urgent but non-emergent condition attributable to a chronic problem or condition that is unlikely to worsen.

### Statistical analysis

A frequency analysis was conducted to identify the status of ED visits. The results of this analysis are presented as frequencies and percentages. A chi-square test was used to examine the distribution of general characteristics according to the frequency of ED use. Multiple logistic regression was performed to examine the factors related to frequent ED use based on odds ratios (ORs) with 95% confidence intervals (CIs). All statistical analyses were performed using SAS software (version 9.4; SAS Institute Inc., Cary, NC, USA). A *p*-value < 0.05 was considered statistically significant.

### Institutional review board waiver statement

This study adhered to the Declaration of Helsinki guidelines and was reviewed by the Severance Hospital Institutional Review Board (IRB number: 4–2021-0491). The requirement of written informed consent was waived by the Severance Hospital Institutional Review Board since the study used secondary anonymized data.

## Results

### Status of ED visits

**Fig. 1:**
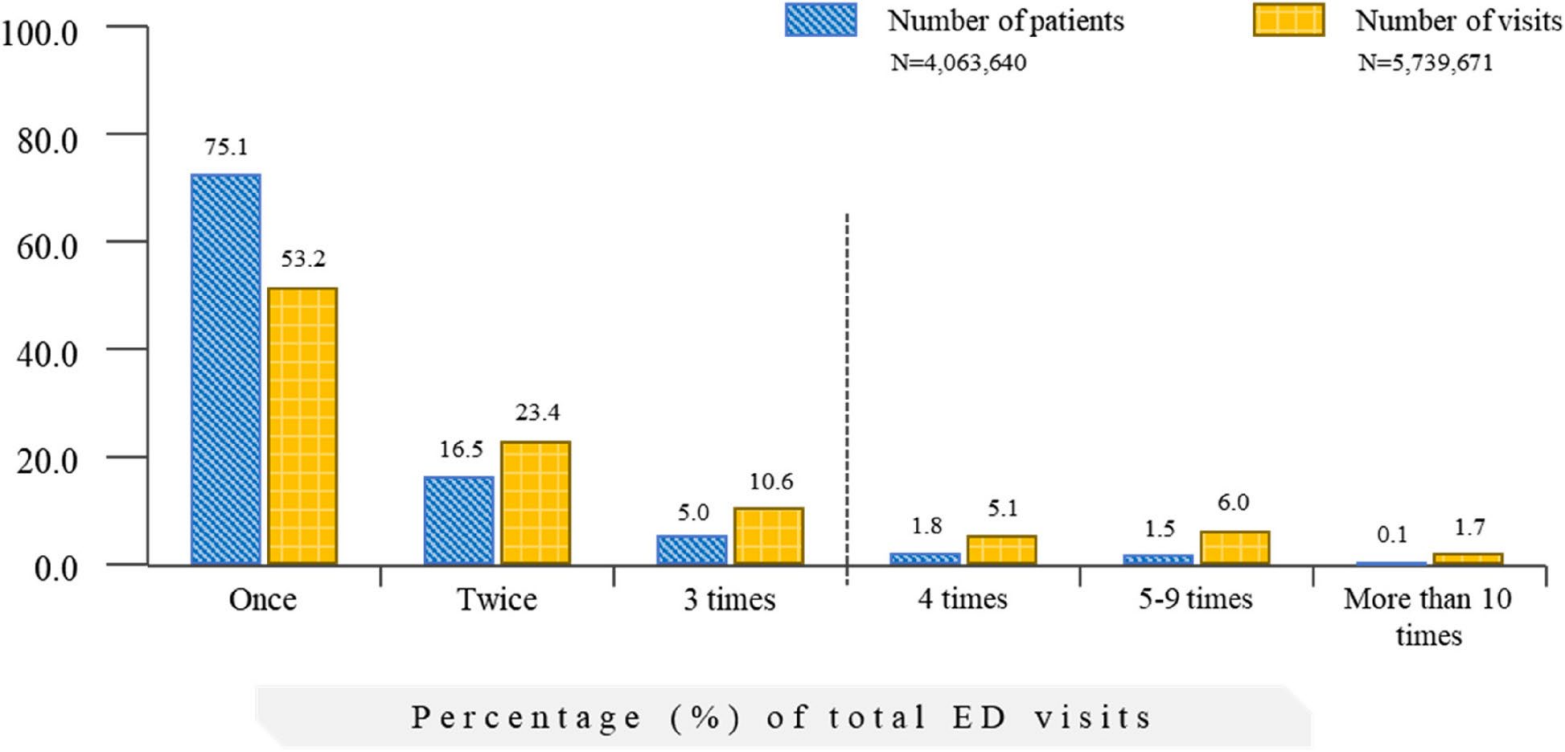
Emergency department visits and percentage of visits

**Fig. 2:**
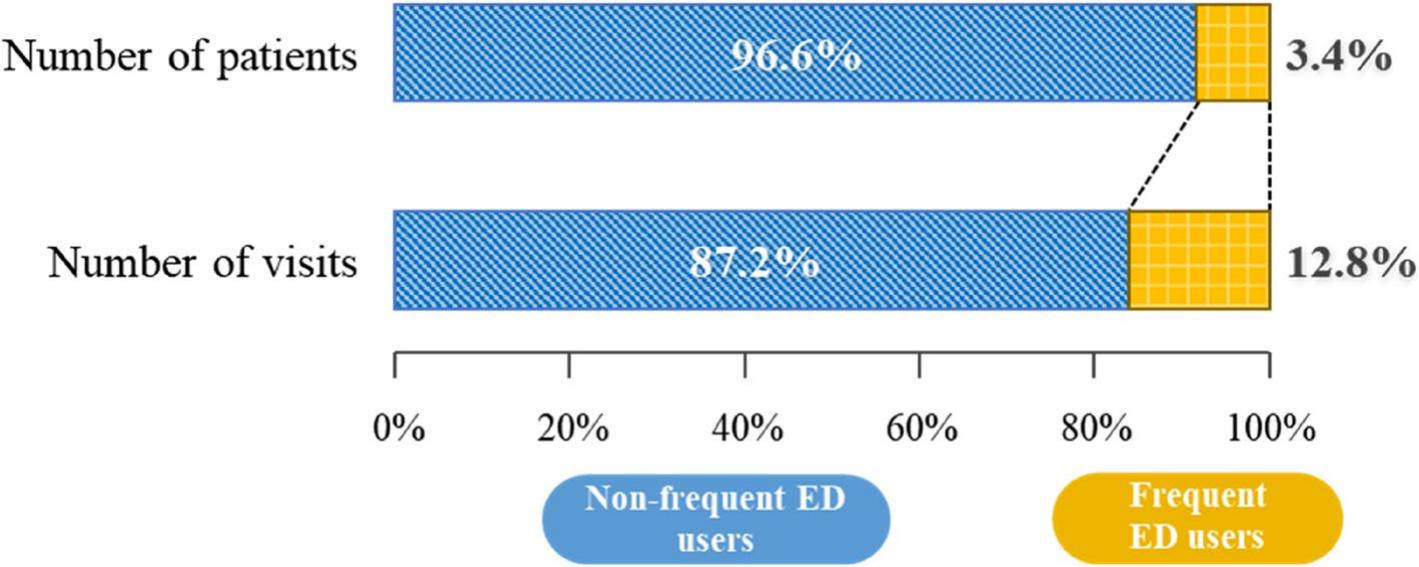
Status of emergency department visits. ED, emergency department

Figure 1 shows the status of ED visits and percentage of visits. Among the total visits, 3,050,671 patients visited the same ED once, which accounted for 75.1% and 53.2% of the total number of ED users and ED visits, respectively. Further, 875,361 patients visited two to three times (total number of visits = 1,953,498 times), which accounted for 21.5% and 34.0% of the total number of ED users and ED visits, respectively. Moreover, 137,608 patients visited the ED four or more times a year (total number of visits = 735,502 times), which accounted for 3.4% and 12.8% of the total number of ED users and ED visits, respectively (Fig. 2).

### Characteristics of patients who visited EDs

The patient characteristics are shown in Table 1. When it is examined according to sex and age groups, there were more male than female patients among frequent ED users. Moreover, patients aged > 70 years (4.7%) were more likely to be frequent ED users, followed by those aged < 9 years (3.9%). Regarding the insurance type, the likelihood of frequent ED use was the highest among patients receiving Medical Aid coverage (7.3%).

**Table 1:** Comparison between frequent and non-frequent ED users

Variables	Total		Frequent ED Users		Non-Frequent ED Users		*p*
	**N**	**%**	**N**	**%**	**N**	**%**	
**Total**	4,063,640	100.0	3,926,032	96.6	137,608	3.4	
**Sex**							< .0001
Male	2,002,672	49.3	1,937,177	96.7	65,495	3.3	
Female	2,060,968	50.7	1,988,855	96.5	72,113	3.5	
**Age (years)**							< .0001
≤ 9	674,626	16.6	648,430	96.1	26,196	3.9	
10–19	292,998	7.2	286,278	97.7	6,720	2.3	
20–29	457,637	11.3	446,438	97.6	11,199	2.4	
30–39	447,022	11.0	435,239	97.4	11,783	2.6	
40–49	459,047	11.3	445,425	97.0	13,622	3.0	
50–59	565,865	13.9	547,863	96.8	18,002	3.2	
60–69	478,823	11.8	461,385	96.4	17,438	3.6	
≥ 70	687,622	16.9	654,974	95.3	32,648	4.7	
**Region**							** < .0001**
Seoul	829,176	20.4	802,376	96.8	26,800	3.2	
Busan	176,214	4.3	171,217	97.2	4,997	2.8	
Daegu	152,200	3.7	148,412	97.5	3,788	2.5	
Incheon	247,928	6.1	238,239	96.1	9,689	3.9	
Gwangju	100,829	2.5	96,637	95.8	4,192	4.2	
Daejeon	105,453	2.6	100,992	95.8	4,461	4.2	
Ulsan	61,968	1.5	60,629	97.8	1,339	2.2	
Sejong	11,554	0.3	11,229	97.2	325	2.8	
Gyeonggi	1,133,162	27.9	1,096,411	96.8	36,751	3.2	
Gangwon	138,037	3.4	133,738	96.9	4,299	3.1	
Chungcheong	342,784	8.4	332,349	97.0	10,435	3.0	
Jeolla	264,711	6.5	253,129	95.6	11,582	4.4	
Gyeongsang	406,565	10.0	391,352	96.3	15,213	3.7	
Jeju	93,059	2.3	89,322	96.0	3,737	4.0	
**Insurance types**							** < .0001**
National Health Insurance	3,622,424	89.1	3,503,781	96.7	118,643	3.3	
Auto insurance	162,973	4.0	159,746	98.0	3,227	2.0	
Industrial accident insurance	8,556	0.2	8,334	97.4	222	2.6	
Medical Aid (Types 1 and 2)	191,451	4.7	177,449	92.7	14,002	7.3	
General insurance	59,510	1.5	58,458	98.2	1,052	1.8	
Other insurance	18,726	0.5	18,264	97.5	462	2.5	
**Residence in underserved emergency Medical Services areas**							** < .0001**
No	3,630,066	89.3	3,506,874	96.6	123,192	3.4	
Yes	433,574	10.7	419,158	96.7	14,416	3.3	
**Number of emergency medical institutions**							** < .0001**
Above the national average	1,306,744	32.2	1,259,050	96.4	47,694	3.6	
Below the national average	2,756,896	67.8	2,666,982	96.7	89,914	3.3	
**Number of beds**							** < .0001**
Above the national average	1,408,186	34.7	1,358,076	96.4	50,110	3.6	
Below the national average	2,655,454	65.3	2,567,956	96.7	87,498	3.3	
**Regional income decile**							** < .0001**
1^st^ quartile (lowest)	405,600	10.0	392,447	96.8	13,153	3.2	
2^nd^ quartile	398,239	9.8	382,396	96.0	15,843	4.0	
3^rd^ quartile	395,879	9.7	382,380	96.6	13,499	3.4	
4^th^ quartile	416,699	10.3	401,957	96.5	14,742	3.5	
5^th^ quartile	428,062	10.5	410,577	95.9	17,485	4.1	
6^th^ quartile	372,942	9.2	359,150	96.3	13,792	3.7	
7^th^ quartile	433,250	10.7	419,650	96.9	13,600	3.1	
8^th^ quartile	371,584	9.1	359,746	96.8	11,838	3.2	
9^th^ quartile	439,894	10.8	427,256	97.1	12,638	2.9	
10^th^ quartile (highest)	401,491	9.9	390,473	97.3	11,018	2.7	
**Cancer**							** < .0001**
No	3,929,064	96.7	3,805,592	96.9	123,472	3.1	
Yes	134,576	3.3	120,440	89.5	14,136	10.5	
**Hypertension**							** < .0001**
No	4,036,995	99.3	3,900,545	96.6	136,450	3.4	
Yes	26,645	0.7	25,487	95.7	1,158	4.3	
**Diabetes**							** < .0001**
No	4,035,224	99.3	3,899,176	96.6	136,048	3.4	
Yes	28,416	0.7	26,856	94.5	1,560	5.5	
**Renal failure**							** < .0001**
No	4,015,410	98.8	3,881,705	96.7	133,705	3.3	
Yes	48,230	1.2	44,327	91.9	3,903	8.1	
**Mental illness**							** < .0001**
No	3,996,542	98.3	3,862,282	96.6	134,260	3.4	
Yes	67,098	1.7	63,750	95.0	3,348	5.0	
**Korean Triage and Acuity Scale**							** < .0001**
Level 1 (Resuscitation)	42,374	1.0	40,698	96.0	1,676	4.0	
Level 2 (Emergent)	216,409	5.3	208,062	96.1	8,347	3.9	
Level 3 (Urgent)	1,493,106	36.7	1,438,032	96.3	55,074	3.7	
Level 4 (Less urgent)	1,967,646	48.4	1,913,001	97.2	54,645	2.8	
Level 5 (Non-urgent)	344,105	8.5	326,239	94.8	17,866	5.2	

Statistically significant at *p*-value < 0.05 (shown in bold)

*ED* Emergency department

When the result was viewed by region, 3.4% and 3.3% of the patients who were non-residents and residents, respectively, of areas lacking emergency medical services were frequent ED users. Regarding the number of emergency medical institutions and beds, there was a higher likelihood of frequent ED visits when the numbers were higher than that of the national average (3.6%) compared with when the number was below. In terms of regional income deciles, among patients living in areas with low-income deciles, 2.7% and 2.9% of patients in the 10^th^ and 9^th^ deciles, respectively, were frequent ED users.

According to the type of chronic diseases, patients with chronic diseases were more likely to be frequent ED users. The highest frequency of ED use was found among patients with cancer (10.5%), hypertension (4.3%), diabetes (5.5%), renal failure (8.1%), and mental illness (5.0%). Additionally, the likelihood of frequent ED use was the highest in patients with level 5 acuity (non-emergency) with the lowest severity.

### Factors related to frequent ED use

Table 2 shows the results of the multiple logistic regression analysis of factors related to frequent ED use. According to the sex and age groups, males and patients aged < 9 years had a higher likelihood of being frequent ED users than females and other age groups. Patients residing in the non-capital areas had a higher range (OR 1.06–1.67) use of ED than those residing in other parts of Korea. Regarding the insurance type, patients with Medical Aid coverage were more likely to be frequent ED users than those with the NHI.

**Table 2:** Multiple logistic regression analysis of factors associated with frequent ED use

Variables	OR	95% CI		*p*
**Sex**				
Female	ref			
Male	1.06	1.05	1.08	** < .0001**
**Age (years)**				
≤ 9	ref			
10–19	0.55	0.54	0.57	** < .0001**
20–29	0.60	0.59	0.61	** < .0001**
30–39	0.64	0.62	0.65	** < .0001**
40–49	0.67	0.65	0.68	** < .0001**
50–59	0.68	0.67	0.69	** < .0001**
60–69	0.74	0.72	0.75	** < .0001**
≥ 70	0.94	0.93	0.96	** < .0001**
**Region**				
Seoul	ref			
Busan	0.96	0.91	1.01	0.1450
Daegu	0.77	0.74	0.80	** < .0001**
Incheon	1.25	1.22	1.29	** < .0001**
Gwangju	1.36	1.28	1.44	** < .0001**
Daejeon	1.33	1.27	1.38	** < .0001**
Ulsan	0.74	0.70	0.79	** < .0001**
Sejong	0.99	0.88	1.11	0.8798
Gyeonggi	1.06	1.04	1.08	** < .0001**
Gangwon	1.04	0.98	1.11	0.1867
Chungcheong	1.16	1.12	1.20	** < .0001**
Jeolla	1.57	1.48	1.65	** < .0001**
Gyeongsang	1.44	1.36	1.51	** < .0001**
Jeju	1.67	1.57	1.78	** < .0001**
**Insurance types**				
National Health Insurance	ref			
Auto insurance	0.70	0.68	0.73	** < .0001**
Industrial accident insurance	0.90	0.79	1.03	0.1312
Medical Aid (Types 1 and 2)	2.12	2.08	2.16	** < .0001**
General insurance	0.60	0.57	0.64	** < .0001**
Other insurance	0.84	0.77	0.92	**0.0002**
**Residence in Underserved Emergency Medical Services areas**				
No	ref			
Yes	0.79	0.77	0.81	** < .0001**
**Number of emergency medical institutions**				
Above the national average	ref			
Below the national average	1.20	1.15	1.25	** < .0001**
**Number of beds**				
Above the national average	ref			
Below the national average	1.04	1.01	1.06	**0.0015**
**Regional income decile**				
1^st^ quartile (lowest)	ref			
2^nd^ quartile	1.25	1.22	1.28	** < .0001**
3^rd^ quartile	1.07	1.05	1.10	** < .0001**
4^th^ quartile	1.12	1.09	1.15	** < .0001**
5^th^ quartile	1.37	1.33	1.40	** < .0001**
6^th^ quartile	1.22	1.19	1.25	** < .0001**
7^th^ quartile	1.01	0.98	1.04	0.4939
8^th^ quartile	1.05	1.02	1.08	**0.0006**
9^th^ quartile	0.95	0.93	0.98	**0.0002**
10^th^ quartile (highest)	0.95	0.92	0.98	**0.0002**
**Cancer**				
No	ref			
Yes	3.33	3.26	3.39	** < .0001**
**Hypertension**				
No	ref			
Yes	0.87	0.82	0.93	** < .0001**
**Diabetes**				
No	ref			
Yes	1.17	1.11	1.24	** < .0001**
**Renal failure**				
No	ref			
Yes	1.91	1.84	1.98	** < .0001**
**Mental illness**				
No	ref			
Yes	1.60	1.54	1.66	** < .0001**
**Korean Triage and Acuity Scale**				
Level 1 (Resuscitation)	ref			
Level 2 (Emergent)	1.04	0.99	1.10	0.1161
Level 3 (Urgent)	1.08	1.03	1.14	**0.0029**
Level 4 (Less urgent)	0.92	0.88	0.97	**0.0013**
Level 5 (Non-urgent)	1.78	1.69	1.88	** < .0001**

Statistically significant at *p*-value < 0.05 (shown in bold)

*OR* Odds ratio, *CI* Confidence interval, *Ref* Reference

In terms of the insurance type, there was a lower likelihood of frequent ED visits among residents than among non-residents of areas lacking emergency medical services. Regarding the number of emergency medical institutions and beds, there was a lower likelihood of frequent ED visits when the numbers were less than the national average.

Regarding the regional income decile, compared with the first income decile, the second, third, fourth, fifth, sixth, seventh, and eighth deciles had higher likelihoods of frequent ED visits, while the ninth and tenth deciles had lower likelihoods of frequent ED visits. Among them, the seventh decile was not statistically significant.

There was relatively high probability of frequent ED visits among patients with cancer, diabetes, renal failure, and mental illness; contrastingly, patients with hypertension had a relatively low probability of frequent ED visits. Regarding the severity classification results, there was higher likelihood of frequent ED visits for level 2 (severe; OR = 1.04), level 3 (emergency), and level 5 (non-emergency) compared with level 1 (resuscitation), while level 4 (quasi-emergency) had lower likelihood of frequent ED visits. Among them, level 2 was not statistically significant.

## Discussion

This study investigated the characteristics and relevant factors related to frequent ED visits (≥ 4 ED visits in a year). We found that among all patients who visited the ED in 2019, 3.4% of them were frequent ED users, which accounted for 12.8% of the total number of ED visits. Among studies conducted outside Korea, frequent ED users accounted for 3.5%–4.5% and 13.9%–18.1% of the total number of ED visitors and visits, respectively [12, 29]. In Korean studies, frequent ED users accounted for 2.7%–3.1% and 11.9%–14.0% of the total number of ED visitors and visits, respectively, which is consistent with our findings [3, 12].

Our findings contribute to the emerging literature on frequent ED users in Korea. First, we observed that patients with Medical Aid coverage showed a higher frequency of ED use than those with NHI coverage, which is consistent with previous reports that frequent ED use has various causes, including relatively low health and access to medical care as well as moral hazards [12, 30]. Patients with Medical Aid coverage have both poor physical health and low socioeconomic status [9]. Since these patients appear to use the ED as an alternative to other sources of primary care, they could use multiple medical resources, including the ED, to address their unmet healthcare needs [16].

Second, Korea has policies for reducing the burden of medical expenses on patients who live in areas without emergency medical services. Health insurance coverage to emergency management fees for non-emergency patients at the same level as emergency patients is applied when these patients visit EDs without symptoms to request emergency medical treatment [31]. Underserved emergency medical service areas are defined as areas where more than 30% of the local population cannot access the local emergency medical center within 30 min or the regional emergency medical center within 1 h. Nonetheless, patients living in areas lacking emergency medical services were less likely to be frequent ED users than patients residing outside such areas. Our findings suggest that living in an area underserved by emergency medical services is a greater limitation to ED use compared with other factors, such as age and income. Furthermore, given the reported low relevance index of areas lacking emergency medical services, there is a need to elucidate ED use in regions to establish emergency medical service provision systems within regions [32] and identify areas lacking emergency medical services. In addition, the research results should be utilized as foundational data for regional health care plans, such as public health care plans for medically vulnerable areas, to devise necessary policies for each region.

Third, we examined the relationship of frequent ED visits with the prevalence of cancer, hypertension, diabetes, renal failure, and mental illness. Patients with cancer were found using the ED frequently, which can be attributed to ED visits for continuous pain relief treatment [12, 33]. Patients with diabetes and renal failure have a high tendency to visit the ED due to acute exacerbation of chronic diseases [3, 17, 34], with patients with renal failure showing a relatively high number of ED visits due to renal dialysis [35]. Chronic diseases are conditions for which adequate ambulatory care can prevent deterioration or complications requiring ED visits or hospitalizations [36]. Primary care should be organized to meet the needs of patients with chronic diseases and a high illness burden [36].

Moreover, patients with mental illness showed a relatively high likelihood of frequent ED visits. This is consistent with previous reports showing that frequent ED use is associated with drug use, alcohol addiction, depression, self-harm, and suicide [3, 17, 37]. We observed no frequent visits among patients with hypertension, which is inconsistent with previous studies [13, 16]. There is an underestimated prevalence of diseases measured by vital signs, including blood pressure, since the NEDIS does not record medical history, such as medical records, which should be considered when interpreting our findings.

Finally, there have been inconsistent reports regarding the acuity of frequent ED users. For example, Vinton et al., [13] Moore et al., [11] and Han et al. [38] reported that the health status of frequent ED users was poor, and the acuity was higher in frequent ED users than in non-frequent ED users. Contrarily, Choe et al., [14] Shin et al., [12] and Uscher-Pines et al. [39] reported no significant difference in acuity between frequent and non-frequent ED users. In our study, there was a higher probability of frequent ED visits in level 5 cases (non-emergency) than in level 1 cases (resuscitation). This suggests that frequent ED visits are contributing to inefficiencies in the medical system, including increasing medical costs and overcrowding, which have been consistently identified in some Korean studies [12, 40].

## Limitations

This study has several limitations. First, since we only used NEDIS data, we only included EDs at the emergency medical center level or higher. However, the NEDIS database is an emergency medical data registration system that is commonly adopted by emergency medical institutions nationwide. It collects medical treatment data from EDs across Korea without restrictions on hospitals and insurance types to enhance the quality of emergency medical services and provide basic data for informing policy and decision-making. Accordingly, it is an excellent source of data with government-managed quality. Second, frequent ED use was defined as a patient using the same ED multiple times since we used patient registration numbers registered for each ED. Third, the prevalence of underlying diseases, including hypertension and diabetes, could have been underestimated since the NEDIS does not provide patient history data, including medical records. Finally, since this was a retrospective cross-sectional study based on 1-year medical data, it cannot demonstrate a causal relationship. Nonetheless, this study has important significance and implications. Specifically, since we used nationwide data recorded in the NEDIS from multiple EDs, it resolves the limitation of existing studies on data from a single ED.

## Conclusion

We found that patients with high medical needs, including those with cancer, chronic disease, and mental illness, were more likely to visit the ED multiple times. Additionally, factors regarding medical access, including low income and disparity in medical resources across regions, were associated with frequent ED use. Future large-scale prospective cohort studies are warranted to establish an efficient emergency medical system because there is variability in geographic, socioeconomic, individual, clinical, and medical-systemic differences. Such studies will achieve a higher level of relevance in emergency medical resource use, the performance of life-saving interventions, admission rates, and mortality in EDs according to the needs of each patient.

## Data Availability

The data that support the findings of this study are available from National Emergency Medical Center under the Ministry of Health and Welfare in Korea, which were used under license for the current study, and so are not publicly available. Informed consent was waived because of the retrospective nature of the study. The datasets which were analyzed during the current study are available from the corresponding author [Tae Hyun Kim] on reasonable request at THKIM@yuhs.ac.

## Abbreviations

ED: Emergency department
NEDIS: National Emergency Department Information System
KTAS: Korean Triage and Acuity Scale
NHI: National Health Insurance
OR: Odds ratio
CI: Confidence interval

## References

[1] Kanzaria HK, Niedzwiecki MJ, Montoy JC, Raven MC, Hsia RY (2017). Persistent frequent emergency department use: core group exhibits extreme levels of use for more than decade. Health Aff (Millwood).

[2] Krieg C, Hudon C, Chouinard MC, Dufour I (2016). Individual predictors of frequent emergency department use: scoping review. BMC Health Serv Res.

[3] Woo JH, Grinspan Z, Shapiro J, Rhee SY (2016). Frequent users of hospital emergency departments in Korea characterized by claims data from the national health insurance: cross sectional study. PLoS ONE.

[4] Sandoval E, Smith S, Walter J, Schuman SA, Olson MP, Striefler R (2010). comparison of frequent and infrequent visitors to an urban emergency department. J Emerg Med.

[5] Lee A, Lau FL, Hazlett CB, Kam CW, Wong P, Wong TW, et al. Measuring the inappropriate utilization of accident and emergency services? Int J Health Care Qual Assur Inc Leadersh Health Serv. 1999;12:287–92. 10.1108/09526869910287558 10724572

[6] Kanzaria HK, Niedzwiecki M, Cawley CL, Chapman C, Sabbagh SH, Riggs E (2019). Frequent emergency department users: focusing solely on medical utilization misses the whole person. Health Aff (Millwood).

[7] Ettinger WH, Casani JA, Coon PJ, Muller DC, Piazza-Appel K (1987). Patterns of use of the emergency department by elderly patients. J Gerontol.

[8] Survey: ED physicians report burnout, desire help for dealing with frequent users. ED Manag. 2011;23(9):104–5. https://pubmed.ncbi.nlm.nih.gov/21916320/. 21916320

[9] Ruger JP, Richter CJ, Spitznagel EL, Lewis LM (2004). Analysis of costs, length of stay, and utilization of emergency department services by frequent users: implications for health policy. Acad Emerg Med.

[10] Colligan EM, Pines JM, Colantuoni E, Howell B, Wolff JL (2016). Risk factors for persistent frequent emergency department use in Medicare beneficiaries. Ann Emerg Med.

[11] Moore L, Deehan, Seed P, Jones R (2009). Characteristics of frequent attenders in an emergency department: analysis of 1-year attendance data. Emerg Med..

[12] Shin TG, Song JW, Song HG, Hong CK (2011). Characteristics of frequent users of emergency department. J Korean Soc Emerg Med.

[13] Vinton DT, Capp R, Rooks SP, Abbott JT, Ginde AA (2014). Frequent users of US emergency departments: characteristics and opportunities for intervention. Emerg Med.

[14] Choe MSP, Seo KS, Kam S, Seo JS, Lee JH, Seol DH (2003). Clinical analysis of frequent attenders of emergency department. J Korean Soc Emerg Med.

[15] Locker TE, Baston S, Mason SM, Nicholl J (2007). Defining frequent use of an urban emergency department. Emerg Med.

[16] Lee JH, Park GJ, Kim SC, Kim H, Lee SW (2020). Characteristics of frequent adult emergency department users: A Korean tertiary hospital observational study. Medicine (Baltimore).

[17] Kim S, Kang H, Cho Y, Lee H, Lee SW, Jeong J (2021). Emergency department utilization and risk factors for mortality in older patients: an analysis of Korean National Emergency Department Information System data. Clin Exp Emerg Med.

[18] Han KS, Kim WY, Kim SJ, Jeong J, Kang H, Lee C (2020). Research for improvement of the national evaluation program for emergency medical center in Korea. J Korean Med Assoc.

[19] Ministry of Health and Welfare (2019). The 3rd National Emergency Medical Plan (2018–2022).

[20] Kim Y, Yeom S, Ryu J, Jeon YJ (2020). Regionalization of emergency medical system and re-establishment of regional emergency medical plan. J Korean Soc Emerg Med.

[21] Lee GW, Lee KW, Jang TC, Kim GM, Seo YW, Ko SH (2018). Relevance of emergency level assessment by the Korean Triage and Acuity Scale for adult patients in a local emergency medical center. J Korean Soc Emerg Med.

[22] Ministry of Health and Welfare. Public Health and Medical Services Act. https://url.kr/qnksbj. Accessed 16 Feb 2022.

[23] Health Insurance Review & Assessment Service. Total Solution for Value-based Healthcare Purchasing HIRA System. https://www.hira.or.kr/eng/ebook/00_Page_img/extra/00.pdf.

[24] Hong JS, Kang HC. Regional differences in treatment requency and casefatality rates in Korean patients with cute myocardial infarction using the Korea national ealth insurance claims database: findings of a large retrospective cohort study. Medicine. 2014;93:e287. 10.1097/MD.0000000000000287 PMC4603128 25526465

[25] Kim H, Kim Y. Factors influencing the use of health services by trauma patients according to insurance type and injury severity score in South Korea: Based on Andersen’s behavioral model. PLoS ONE. 2020;15:e0238258. 10.1371/journal.pone.0238258 PMC7451573 32853228

[26] Industrial Accident Compensation Insurance Act. https://elaw.klri.re.kr/kor_service/lawView.do?hseq=51148&lang=ENG.

[27] Jo M, Oh H, Jang SY (2021). The effect of residence in underserved emergency medical services areas on awareness of myocardial infarction symptoms in Korea. J Health Info Stat.

[28] Park J, Lim T (2017). Korean triage and acuity scale (KTAS). J Korean Soc Emerg Med.

[29] Hansagi H, Olsson M, Sjoberg S, Tomson Y, Göransson S (2001). Frequent use of the hospital emergency department is indicative of high use of other health care services. Ann Emerg Med.

[30] Kim S, Lee J (2017). Utilization of emergency medical services according to the medical aid benefit. Health Serv Manage Res.

[31] Kim BK. Applying health insurance to emergency rooms and non-emergency patients in areas vulnerable to emergency medical care. Yonhap News. 2015. https://www.yna.co.kr/view/AKR20151126192500017. Accessed 19 May 2022.

[32] Oh M, Jeon B, Lee J, Jeong T, Heo T (2019). Inflow and outflow type analysis of emergency department patients of the Honam region. J Korean Soc Emerg Med.

[33] Acosta AM, da Silva Lima MAD (2015). Frequent users of emergency services: associated factors and reasons for seeking care. Rev Lat Am Enfermagem.

[34] Ustulin M, Woo J, Woo JT, Rhee SY (2018). Characteristics of frequent emergency department users with type 2 diabetes mellitus in Korea. J Diabetes Investig.

[35] Komenda P, Tangri N, Klajncar E, Eng A, Di Nella M, Hiebert B (2018). Patterns of emergency department utilization by patients on chronic dialysis: population-based study. PLoS ONE.

[36] Hudon C, Courteau J, Krieg C, Vanasse A (2017). Factors associated with chronic frequent emergency department utilization in a population with diabetes living in metropolitan areas: a population-based retrospective cohort study. BMC Health Serv Res.

[37] Behr JG, Diaz R (2016). Emergency department frequent utilization for non-emergent presentments: Results from a regional urban trauma center study. PLoS ONE.

[38] Han JO, Kang KH, Yim J (2017). The usual source of healthcare and frequent visits to emergency departments. J Korean Soc Emerg Med.

[39] Uscher-Pines L, Pines J, Kellermann A, Gillen E, Mehrotra A (2013). Emergency department visits for nonurgent conditions: Systematic literature review. Am J Manag Care.

[40] Lee I (2020). The limit of emergency medical services: emergency services public goods. HIRA Res.

